# Multi-substrate biodegradation of chlorophenols by defined microbial consortium

**DOI:** 10.1007/s13205-016-0511-x

**Published:** 2016-09-02

**Authors:** Bhishma P. Patel, Arvind Kumar

**Affiliations:** 1 Value Addition, Research and Development Department, National Innovation Foundation-India, Satellite Complex, Jodhpur Tekra, Ahmedabad, 380015 Gujarat India; 2 Environmental Pollution Abatement Lab, Chemical Engineering Department, National Institute of Technology, Rourkela, 769008 Odisha India

**Keywords:** Monochlorophenol, 2,4-Dichlorophenol, Biodegradation, Multi-substrate degradation, Kinetics

## Abstract

In the present study, a defined mixed microbial consortium was investigated for their ability to utilize three different monochlorophenols (MCPs) and 2,4-DCP individually and in the mixture. None of the individual strains were able to utilize 3-CP and 4-CP, but when they were mixed to form defined consortium, they have shown great potential and degradation of high concentration of 3-CP and 4-CP. Spectrophotometric analysis of metabolites during MCPs degradation establishes the presence of 2-chloromaleylacetate. Multi-substrate degradation study of 2,4-DCP in the presence of three MCPs showed the great prospect of microbial consortium for in situ bioremediation. During multi-substrate degradation, the biodegradation rate (mg L^−1^ day^−1^) was observed in the order of 2,4-DCP > 2CP > 3CP > 4CP. Biodegradation kinetic of three MCPs using Andrew’s model showed maximum removal rate (*R*_m_) of 2.78, 0.91, 1.82 mg L^−1^ h^−1^ for 2-CP, 3-CP and 4-CP, respectively.

## Introduction

Chlorophenols are regarded as a serious menace to the environment due to their widespread occurrence and toxicity to human and other flora and fauna (Olaniran and Igbinosa 2011; Snyder et al. 2006). The primary sources of MCPs are the chlorination of drinking water and wastewater, degradation of chloride containing pesticides and higher chlorophenols (ATSDR 1999). Other sources of MCPs and DCPs contamination are an industrial discharge of pulp and paper, dye, leather and tanning industries (Olaniran and Igbinosa 2011; Marihal et al. 2009; Field and Sierra-Alvarez 2008; Wang et al. 2014). Chlorophenols have a detrimental effect on the environment due to their physicochemical properties, which results in higher persistence and subsequent bioaccumulation (Olaniran and Igbinosa 2011; Marihal et al. 2009). They have been classified as a priority pollutant by the US Environmental Protection Agency (ATSDR 2015). Different physicochemical treatments such as oxidation, solvent extraction, photodegradation, and adsorption are only transforming the compounds from one phase to another. High cost, toxic by-product yield, low energy efficiency limits the employment of physicochemical methods (Herrera et al. 2008; Olaniran and Igbinosa 2011). On the contrary, biological treatment is a potential substitute with promising removal efficiency and complete mineralization of the chlorophenols. It is an eco-friendly and energy-efficient treatment with assuring future (Field and Sierra-Alvarez 2008; Karigar and Rao 2011).

There exist various reports on biodegradation of chlorophenols using pure as well as mixed microorganisms (Baggi et al. 2002; Nordin et al. 2005; El-Sayed et al. 2009; Solyanikova and Golovleva 2004; Farrell and Quilty 1999; Patel and Kumar 2016b). A cleavage of aromatic ring and removal of chloride ions are two important steps necessary for complete mineralization of chlorophenol compounds (Häggblom 1990). Aerobic biodegradation of MCPs mainly follows two pathways, *ortho*- and *meta*-fission. The first step in the biodegradation of MCPs is their transformation into chlorocatechol (CC). 2-Chlorophenol (2-CP) and 3-chlorophenol (3-CP) are being converted to 3-chlorocatechol (3-CC) while, 4-chlorophenol (4-CP) is converted to 4-chlorocatechol (4-CC) (Arora and Bae 2014; Häggblom 1990). The second step is the ring cleavage of chlorocatechol by dioxygenases. The aromatic ring cleavage of CC occurs via either *ortho*- or *meta*-pathway (Veenagayathri and Vasudevan 2011). Chloroaromatic compounds degraded via *ortho*-pathway catalyzed by enzyme catechol 1,2-dioxygenase (Farrell and Quilty 1999). While the catechol 2,3-dioxygenase are responsible for the *meta*-cleavage of CC. The *meta*-cleavage of 3-CC results in the dead-end pathway due to the generation of suicide or dead-end metabolites (Schmidt et al. 1983; Bartels et al. 1984; Klecka and Gibson 1981). The *meta*-cleavage of 4-CC results in the production of 5-chloro-2-hydroxymuconic semialdehyde (5-CHMS) which has been reported as dead-end metabolite (Wieser et al. 1994; Arora and Bae 2014). But recent studies have shown the further degradation of 5-CHMS, indicating complete degradation of 4-CP via *meta*-fission pathway (Westmeier and Rehm 1987; Farrell and Quilty 1999; El-Sayed et al. 2009).

Co-metabolism of chlorophenols is important in understanding the in situ bioremediation, synergetic of the biodegradation and multi-substrate degradation process that has been encountered in the environment. Carbon and nitrogen sources such as glucose, sodium acetate, peptone, yeast extract were reported to enhance the biodegradation of chlorophenols (Murialdo et al. 2003; Shen et al. 2005; Sahinkaya and Dilek 2006a, b; Patel and Kumar 2016a). However, this would increase the cost of the treatment. Other studies recommended the use of lower toxic compounds such as lower phenolic compounds for increased degradation of higher toxic compounds (Wang et al. 2014; Durruty et al. 2011; Tobajas et al. 2012; De Los Cobos-Vasconcelos et al. 2006). The degradation of higher toxic recalcitrant compounds has been found to increase in the presence of lower toxic compounds due to their structural similarity, increased biomass growth and enzymes induced by lower toxic compounds facilitate the degradation of higher toxic compounds (Durruty et al. 2011; Alexander 1999). However, converse effects were also reported in the literature.

In the present study, the biodegradation of three monochlorophenols (2-CP, 3-CP, and 4-CP) by the defined mixed microbial consortium was studied. The effects of initial substrate concentration, biodegradation kinetic and intermediate metabolites were studied. In addition, the multi-substrate degradation by defined mixed consortium was analyzed to study the effect of the presence of three MCPs on the biodegradation of 2,4-DCP in the various combination.

## Materials and methods

### Microbial consortium

The defined mixed microbial consortium used in the study was prepared by mixing four different microbial strains in equal proportion. All the four strains used in this study were previously isolated from sludge, and soil samples collected from the dye industries effluent treatment plant, Gujarat, India. All the four strains were acclimated to 2,4-DCP using mineral salt medium (MSM) (composition mentioned in “Biodegradation study of MCPs”) containing peptone (1–0.2 g L^−1^) and 2,4-DCP (up to 200 mg L^−1^) and incubated at 30 °C and 120 rpm in a rotary shaker for a period of 4–6 months under aerobic condition. During the acclimatization period, culture is transferred to fresh MSM every 15 days with increasing concentration of 2,4-DCP. The 2,4-DCP-degrading bacterial strains were isolated from the final acclimatized culture using serial dilution technique and purified by repeated streaking on MSM agar plates containing 1.5 % agar and 50 mg L^−1^ 2,4-DCP. Based on the 16s rDNA sequence analysis, the four strains were identified as *Bacillus endophyticus* strain CP1R (Genbank Accession no.: KM259919), *Bacillus cereus* strain 3YS (KM522855), *Kocuria rhizophila* strain 11Y (KM522854), and *Pseudomonas aeruginosa* strain GF (KM259920). All the strains were maintained on MSM agar with 1 g L^−1^ peptone, 50 mg L^−1^ 2,4-DCP and 1.5 % agar, pH 7.0 ± 0.1.

### Chemicals and reagents

Loba Chemie, India supplied analytical grade 2-CP, 3-CP, 4-CP and 2,4-DCP (purity 98 %). The stock solution of all the chlorophenol compounds is prepared in 0.02 M NaOH and pH was adjusted to 7.4 ± 0.2 by 1 M orthophosphoric acid. All other inorganic chemicals used in the experiments were of analytical grade and obtained from Merck, India. HPLC-grade methanol and hydrochloric acid were obtained from Hi-media, India for HPLC analysis.

### Biodegradation study of MCPs

Batch study for biodegradation of 2-CP, 3-CP and 4-CP was performed in Erlenmeyer flasks (250 mL) containing 50 mL MSM (modified DSMZ-465) having composition of (g L^−1^): Na_2_HPO_4_·2H_2_O 3.5, KH_2_PO_4_ 1, (NH_4_)_2_SO_4_ 0.5, MgCl_2_·6H_2_O 0.1, NaNO_3_0.05 and 1 mL of trace element solution having composition of (g L^−1^): EDTA 0.5, FeSO_4_·7H_2_O 0.2, CuCl_2_·2H_2_O 0.001, ZnSO_4_·7H_2_O 0.01, MnCl_2_·4H_2_O 0.003, CoCl_2_·6H_2_O 0.02, H_3_BO_3_ 0.03, Na_2_MoO_4_·2H_2_O 0.003. 2-CP (50–400 mg L^−1^), 3-CP (50–400 mg L^−1^) and 4-CP (50–600 mg L^−1^) were added to the sterilized medium by filter sterilizing using 0.22 µm syringe filter. For inoculation purpose, all the four strains were cultured in N-broth with 50 mg L^−1^ 2,4-DCP for 24–36 h so that OD value in each culture reaches the same value, i.e., 1.0 at 600 nm. These four cultures (one mL each) were added to the experimental flask for biodegradation study. The blank flask was set up without microorganism to check the abiotic loss of MCPs in the medium. All the experiments were performed in duplicate, and the mean values of the same are presented. Samples were withdrawn at fixed interval for analysis of biomass, residual chlorophenol, and intermediate products.

### Multi-substrate degradation of MCPs and 2,4-DCP

Multi-substrate degradation study was carried out to analyze biodegradation of 2,4-DCP in the presence of MCPs and ability of the defined mixed consortium to simultaneously utilize different chlorophenols. The study was performed in Erlenmeyer flask (250 mL) with 50 mL MSM having a composition as mentioned above (“Biodegradation study of MCPs”). A different combination of 2,4-DCP and MCPs added to the flask is mentioned in Table 1. Samples were withdrawn at regular intervals for HPLC analysis of residual chlorophenol concentration.

**Table 1:** A different combination of 2,4-DCP and MCPs used in the co-metabolic study

Compounds	DCP	2CP	3CP	4CP	Total CP
DCP	50	–	–	–	50
DCP + 2CP	50	25	–	–	100
DCP + 3CP	50	–	25	–	100
DCP + 4CP	50	–	–	25	100
DCP + 2CP + 3CP	50	25	25	–	100
DCP + 2CP + 4CP	50	25	–	25	100
DCP + 3CP + 4CP	50	–	25	25	100
DCP + 2CP + 3CP + 4CP	50	17	17	17	103

All the concentration values are in mg L^−1^

### Kinetic study

Kinetics of 2-CP, 3-CP, and 4-CP biodegradation was performed in batch. Chlorophenols are toxic and have inhibition effect on microorganism at a higher concentration. So the Haldane/Andrew’s substrate inhibition model was used to calculate the biokinetic parameters for degradation of MCPs (Andrews 1968; Kargi and Eker 2005).

1 $$R_{\text{s}} = \frac{{R_{\text{m}} S}}{{K_{\text{s}} + S}}\frac{{K_{\text{si}} }}{{K_{\text{si}} + S}} = \frac{{R_{\text{m}} }}{{\left( {1 + \frac{{K_{\text{s}} }}{S}} \right)\left( {1 + \frac{S}{{K_{\text{si}} }}} \right)}}$$

where *R*_s_ and *R*_m_ are the actual and maximum rate of CP degradation (mg CP L^−1^ h^−1^); *S* is the initial CP concentration (mg L^−1^); *K*_s_ is the saturation constant (mg L^−1^); *K*_si_ is the inhibition constant (mg L^−1^).

For lower substrate concentration, the inhibition constant can be neglected. Hence, the above equation becomes

2 $$R_{\text{s}} = \frac{{R_{\text{m}} S}}{{K_{\text{s}} + S}}$$

In the linear form

3 $$\frac{1}{{R_{\text{s}} }} = \frac{1}{{R_{\text{m}} }} + \frac{{K_{\text{s}} }}{{R_{\text{m}} }}\frac{1}{S}$$

As chlorophenol is a toxic compound, there is a critical substrate concentration above which the removal rate decreases (Tomei et al. 2004; Sahinkaya and Dilek 2007). Critical substrate concentration can be obtained by taking the derivation of Eq. 1 with respect to *S*.

4 $$\frac{{{\text{d}}R_{\text{s}} }}{{{\text{d}}S}} = 0$$

Solving for *S*_max_ or *S* ^*^,

5 $$S_{ \hbox{max} } = \sqrt {K_{\text{s}} K_{\text{i}} }$$

*S*_max_ = critical substrate concentration after which removal rate decreases. The biokinetic constants were determined using the MATLAB 6.5. The software used the sum square error estimation function for solving the equation.

### Analytical methods

Biomass concentration was determined by measuring optical density at 600 nm by UV–Visible spectrophotometer (Shimadzu UV-1800, Japan) (Ziagova and Liakopoulou-Kyriakides 2007). The residual concentrations of 2,4-DCP and MCPs were determined by HPLC system (Jasco, US) coupled with MD-2015 photodiode array detector and 2080 plus isocratic pump. The 1 mL sample was centrifuged at 10,000 rpm for 12 min and supernatant was filtered through 0.22 µm filter before analysis. The sample was acidified to pH 2 with 1 M hydrochloric acid before analysis. The column used was Agilent TC-C18 (25 mm × 4.6 mm); the sample was eluted at a flow rate of 0.75 mL/min with mobile phase consisting of methanol:water (80:20); detection wavelength is set to 280 nm (Sahinkaya and Dilek 2007; Karn et al. 2010). 2-CP, 3-CP, and 4-CP were also analyzed using UV–Visible spectrophotometer at 273, 273 and 279 nm wavelengths, respectively (Goswami et al. 2002).

The presence of 5-chloro-2-hydroxymuconic semialdehyde (5-CHMS) was determined at 380 nm UV–Visible spectrophotometer (Shimadzu UV-1800, Japan) (Farrell and Quilty 1999).

Chlorocatechol was analyzed using the method of Arnow (Arnow 1937). The samples were centrifuged at 10,000 rpm for 10 min to removal cells. The supernatant (0.5 mL) was treated with 0.5 N HCL (0.5 mL). After mixing, 0.5 mL of nitrite molybdate reagent was added to it and mixed resulting in a yellow color. Nitrite molybdate reagent was prepared by dissolving 10 gm of sodium nitrite and 10 gm of sodium molybdate in 100 mL distilled water. After mixing, 0.5 mL of 1 N NaOH was added resulting in red color. Following mixing, the absorbance was measured at 510 nm.

## Results and discussions

### Biodegradation of monochlorophenols

**Fig. 1:**
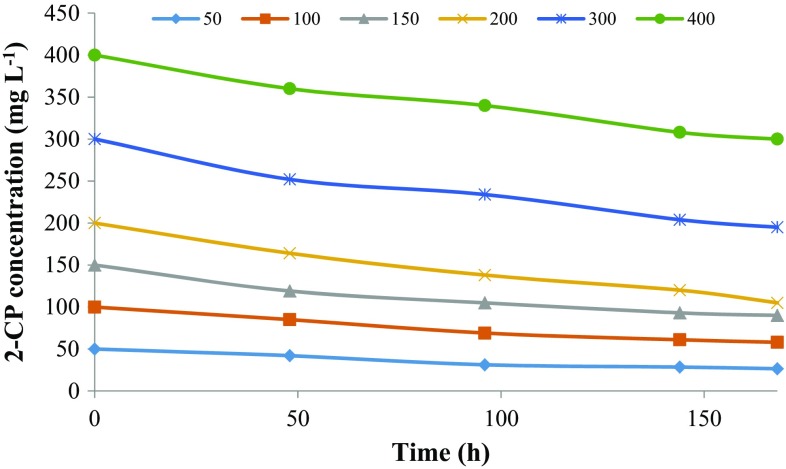
Biodegradation profile of 2-CP with time at different initial substrate concentrations

**Fig. 2:**
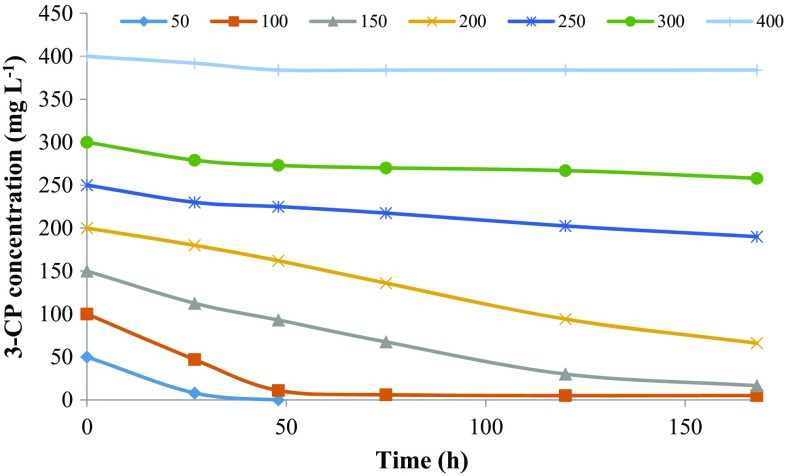
Biodegradation profile of 3-CP with time at different initial substrate concentrations

**Fig. 3:**
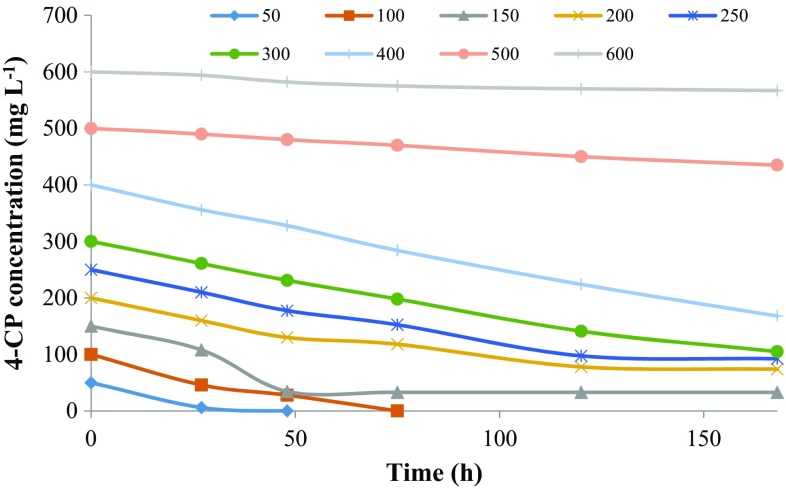
Biodegradation profile of 4-CP with time at different initial substrate concentrations

Biodegradation of 2-CP, 3-CP, and 4-CP at different initial concentration was carried out by the defined consortium for 168 h, and their degradation profiles are shown in Figs. 1, 2 and 3, respectively. The abiotic loss due to evaporation for 2-CP was 4–15 % while for 3-CP and 4-CP, the abiotic loss observed was negligible (2–3 %). The abiotic losses observed were adjusted in the presented data. The mixed consortium was not able to achieve complete degradation of 2-CP up to 400 mg L^−1^ of initial substrate concentration within 168 h. The biodegradation obtained was in the range of 25–47 % for an initial substrate concentration of 50–400 mg L^−1^ within 168 h. The percentage degradation was decreased with increasing substrate concentration. The abiotic loss due to evaporation was observed to increase with initial substrate concentration. The reason for incomplete degradation of 2-CP by the mixed consortium at lower concentration was not clear.

In the case of 3-CP, the microorganisms had completely degraded 50 mg L^−1^ within 30 h. While for 100 and 150 mg L^−1^ of initial 3-CP concentration, the degradation obtained was 95 and 89 % within 120 and 168 h, respectively. After 200 mg L^−1^ of 3-CP, the inhibition effect was more persistent resulting in a decreased removal rate and an increased lag phase. The microorganisms showed 67, 24, 14 and 4 % removal of 200, 250, 300 and 400 mg L^−1^ of 3-CP, respectively, within 168 h.

The microorganisms showed complete removal of 50 and 100 mg L^−1^ of 4-CP within 32 and 72 h, respectively, while it had removed 77 % of 150 mg L^−1^ within 48 h. The percentage removal obtained for 200, 250, 300 and 400 mg L^−1^ of initial 4-CP was 63, 63, 65 and 58 % within 168 h, respectively. The inhibition effect was more persistent at 500 and 600 mg L^−1^ of 4-CP for which the consortium showed only 13 and 5.5 % degradation within 168 h, respectively. It was found that the removal of 3-CP and 4-CP decreased and became constant after a period due to the inhibition effect caused by the accumulation of metabolites.

**Fig. 4:**
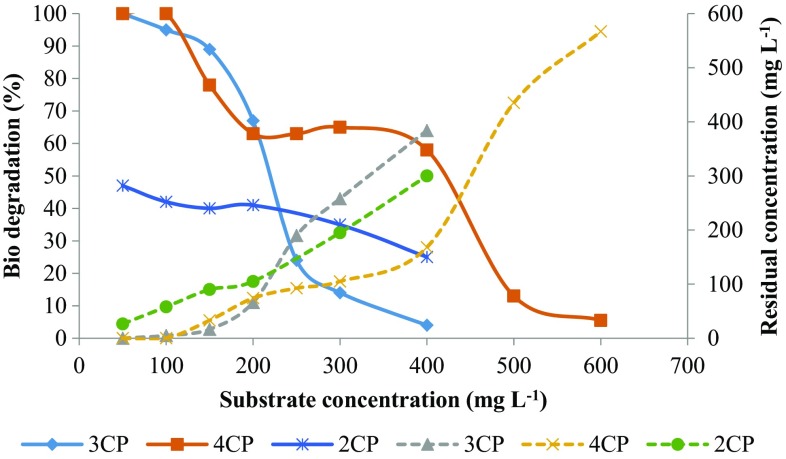
Effect of initial MCPs concentration on biodegradation (%) and residual MCPs concentration

**Fig. 5:**
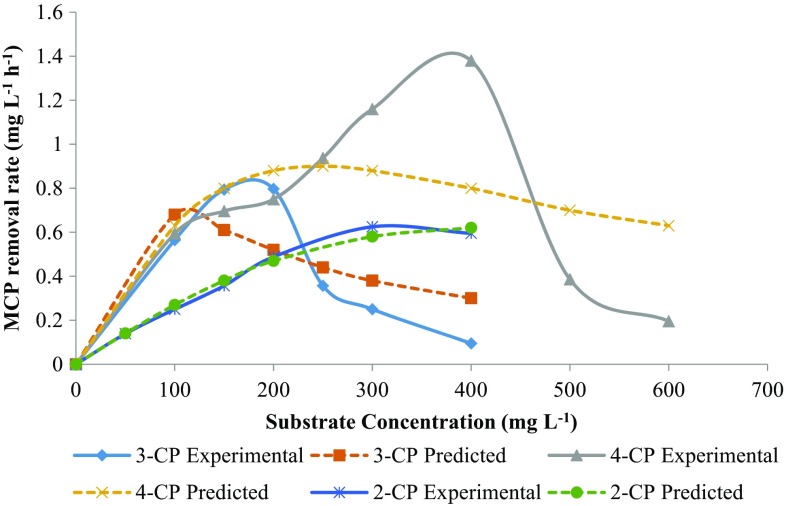
Effect of initial substrate concentration on the MCPs removal rate

Figure 4 shows the effect of initial substrate concentration on the percent biodegradation and final residual concentration of MCPs. 3-CP showed higher biodegradation (%) at a low concentration as compared to 4-CP, which was diminished greatly at higher concentration while 2-CP and 4-CP showed a higher rate of biodegradation (%) at a high concentration. Figure 5 shows the effect of initial substrate concentration on the removal rate of MCPs. For 4-CP, the removal rate was increased with substrate concentration up to 400 mg L^−1^ and then diminished rapidly for 500 and 600 mg L^−1^ of initial substrate concentration. For 3-CP, the removal rate was increased with substrate concentration up to 150 mg L^−1^ and then decreased afterward showing the inhibition effect on microorganism at a higher concentration. While for 2-CP, the removal rate was increased up to 300 mg L^−1^ and then decreased slightly at 400 mg L^−1^. For 3-CP, the inhibition stage was reached at a lower concentration as compared to 2-CP and 4-CP. The toxicity of the MCPs is in the order of 2CP < 4CP < 3CP. The removal of chloride residue at *ortho*-position requires less energy while the *meta*-position is more energy demanding due to steric hindrance, indicating the higher toxicity of 3-CP as compare to other MCPs (Papazi and Kotzabasis 2013).

Table 2 summarizes the biodegradation kinetic parameters for MCPs calculated using Andrew’s substrate inhibition model (Eq. 1). The maximum removal rate obtained was 2.78, 1.82, and 0.91 mg CP L^−1^ h^−1^ for 2-CP, 4-CP and 3-CP, respectively. The inhibition constant is an important biokinetic parameter that determines the inhibition effect of the compound on a microorganism. The higher the inhibition constant, lower the inhibition effect on the microorganism. The inhibition constant (*K*_i_) obtained for MCPs was 1061.6, 323.2, 189.46 mg L^−1^ for 2-CP, 4-CP and 3-CP, respectively. The higher inhibition constant for 2-CP indicates less inhibition effect on microorganism compared to 3-CP and 4-CP. 3-CP has the lowest value for inhibition constant indicating higher toxicity on the mixed consortium. The half-saturation constant (*K*s) obtained for 2-CP, 3-CP and 4-CP was 956, 46.57, and 225.68, respectively. The lower value of half-saturation constant (*K*s) for 3-CP indicates that the maximum removal rate was achieved at lower concentration compared to 2-CP and 4-CP as shown in Fig. 5. The kinetic constants obtained for 3-CP and 4-CP were in accordance with the literature. The critical substrate concentration (Eq. 5), after which removal rate decreases, obtained was 1007.41, 93.9 and 270 mg L^−1^ for 2-CP, 3-CP, and 4-CP, respectively.

**Table 2:** Biodegradation kinetic constants obtained for 3-CP and 4-CP using Andrew’s model

Compounds	*K*_S_ (mg L^−1^)	*K*_i_ (mg L^−1^)	*R*_m_ (mg L^−1^ h^−1^)	*R* ^2^
2-CP	956	1061.6	2.78	0.99
3-CP	46.57	189.46	0.91	0.93
4-CP	225.68	323.2	1.82	0.95

### Metabolites

**Fig. 6:**
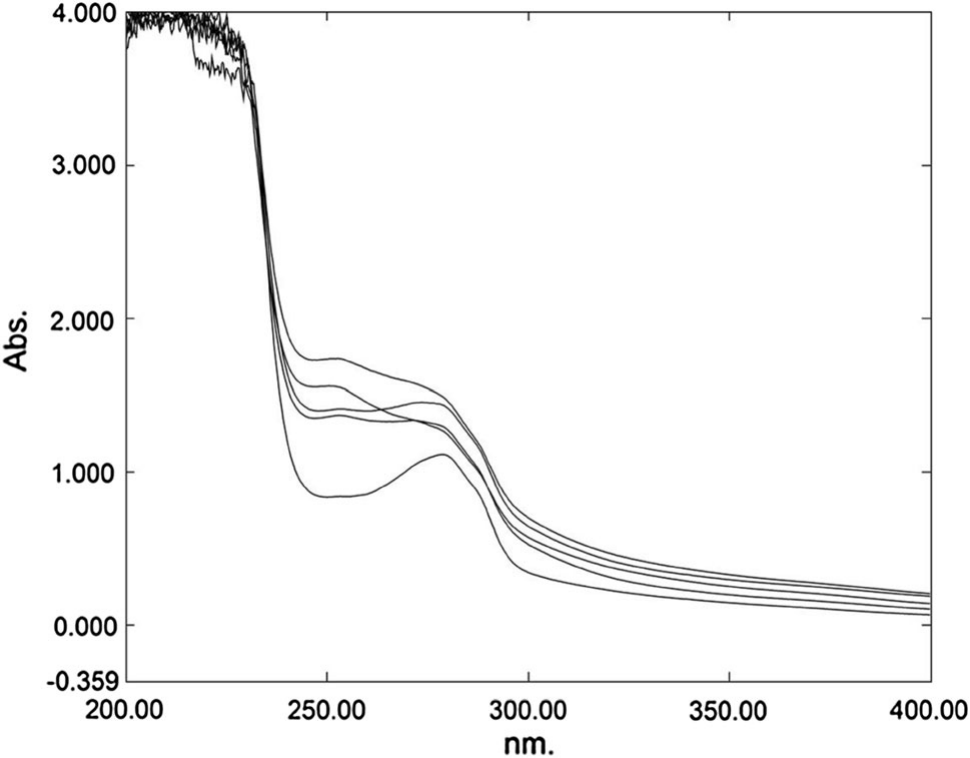
Spectrophotometric analysis of biodegradation of 4-CP (50 mg L^−1^) by the mixed consortium. The absorbance of 4-CP was at 279 nm and the second peak appearing at 253 nm is related to 2-chloromaleylacetate as degradation progress. The *lower* and *upper lines* show degradation of 4-CP at 0 and 168 h, respectively

HPLC and spectroscopic analysis showed the presence of different intermediate metabolites during the biodegradation. There were no residual intermediate products detected during the degradation of 2-CP. During the biodegradation of both 3-CP and 4-CP, one characteristic peak at 251–253 nm was observed. According to the literature, this peak was related to 2-chloromaleylacetate (Louie et al. 2002). This peak was observed during biodegradation for a higher concentration of 3-CP and 4-CP. Figure 6 shows the spectrophotometric analysis of biodegradation of 4-CP (50 mg L^−1^) by the mixed consortium. From the figure, it was observed that as degradation progresses, a new peak at 253 nm appears. This peak for 2-chloromaleylacetate disappeared at the end of the degradation indicating the complete mineralization of the MCPs. In the case of 4-CP, at higher concentration, the absorbance at 370–375 nm shows the presence of trace amount of 2-hydroxymuconic semialdehyde.

### Multi-substrate degradation study

**Fig. 7:**
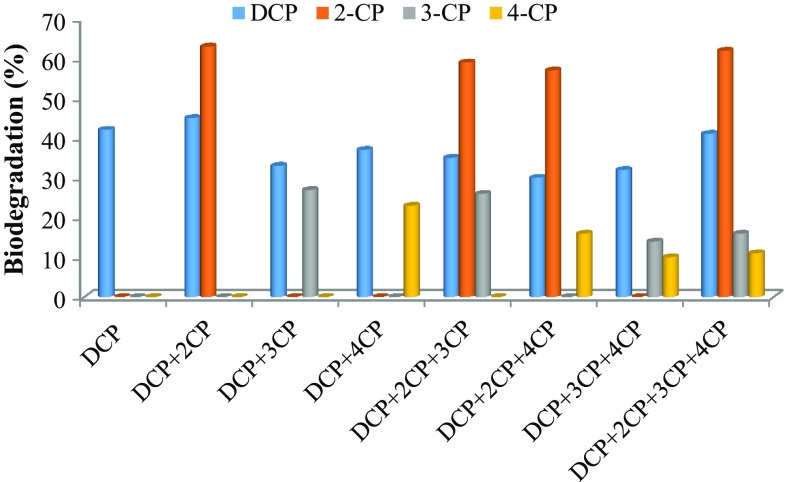
Biodegradation (%) obtained for 2,4-DCP and monochlorophenols during the co-metabolic study

**Fig. 8:**
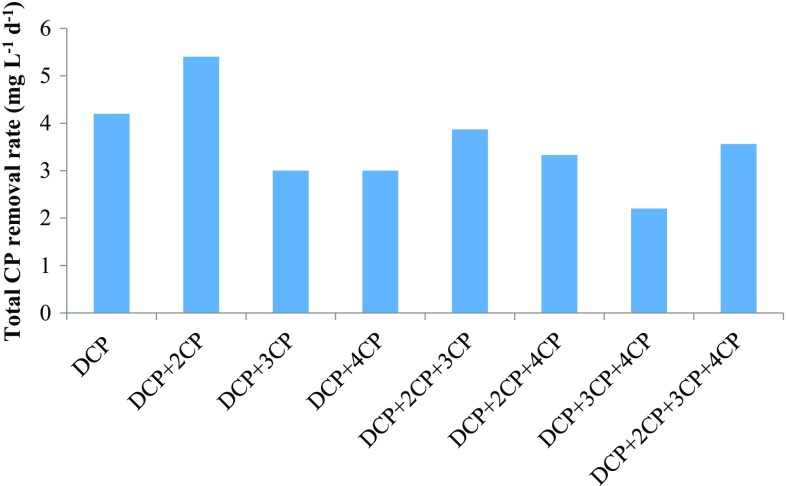
Total chlorophenol removal rate obtained for 2,4-DCP and MCPs during the co-metabolic study

**Fig. 9:**
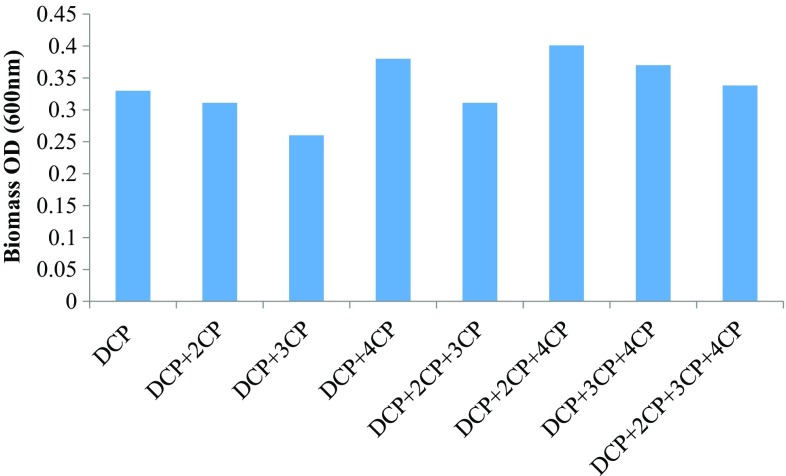
Maximum biomass (OD) achieved by the mixed consortium during the co-metabolic study

In situ bioremediation of chlorophenols is complex and requires the understanding of microorganism’s ability to utilize different chlorophenol congeners simultaneously. It has been reported that apart from various environmental factors such as pH, temperature, and nutrient, the presence of other toxic compounds and the biodegradation products of parent compounds have an effect on biodegradation. The presence of nutrient and lower phenolic compounds has positive as well as negative effects on the degradation of higher chlorophenols depending on the molecular structure, enzyme expression, interaction between growth and non-growth substrate and presence of metabolites (Alexander 1999; Durruty et al. 2011). 2,4-DCP has two chloride ion substitutions, one at *ortho* and other at the *para*-position while monochlorophenols have three different congeners, *ortho*-(2-CP), *meta*-(3-CP) and *para*-(4-CP), with one chloride substitution at different positions. Effect of 2-CP and 4-CP on 2,4-DCP biodegradation is important due to structural similarity between them regarding the position of chloride ion. The biodegradation of 2,4-DCP in the presence of three different MCPs, alone and in the mixture, was analyzed, and the results are shown in Fig. 7. The total chlorophenol removal rate and biomass (maximum OD at 600 nm) obtained are shown in Figs. 8 and 9, respectively.

First, the effect of the presence of one MCP on the degradation of 2,4-DCP was studied. In the binary mixture, the mixed consortium showed 45, 33, 37 % degradation of 2,4-DCP in the presence of 2-CP, 3-CP and 4-CP, respectively. While the degradation obtained for 2-CP, 3-CP and 4-CP was 63, 27 and 23 %, respectively. The mixed consortium showed 42 % degradation of 2,4-DCP alone within 240 h. In the presence of 3-CP and 4-CP, the biodegradation of 2,4-DCP was decreased as compared to DCP alone, while a slight increase in 2,4-DCP degradation observed in the presence of 2-CP. However, the total chlorophenol degradation was higher for 2CP + 2,4-DCP mixture (54 %) compared to DCP alone (42 %) and other binary mixture, i.e., 3CP + 2,4-DCP and 4CP + 2,4-DCP (30 % in both cases).

In the next study, the effect of the presence of two different MCPs on the biodegradation of 2,4-DCP was evaluated. In the tertiary mixture, the highest biodegradation obtained for 2,4-DCP was 35 % in the presence of 2CP + 3CP. The biodegradation of 2,4-DCP was decreased to 30 and 32 % in the presence of other MCPs binary mixtures as shown in Fig. 7. Biodegradation of 2-CP was higher in the tertiary mixture as compared to other chlorophenols. The biodegradation rate (mg L^−1^ day^−1^) observed for chlorophenols in the tertiary mixture was in the order of DCP > 2CP > 3CP > 4CP.

In the quaternary mixture of 2,4-DCP and MCPs, the effect of the presence of all three MCPs on DCP degradation was studied. 2,4-DCP showed 41 % degradation which was higher as compared to the tertiary mixture, while the degradation for MCPs obtained was 62, 16, and 11 % for 2-CP, 3-CP and 4-CP, respectively. The degradation obtained for 2,4-DCP was nearly equal to that obtained for 2,4-DCP alone. The biodegradation (mg L^−1^ day^−1^) occurred in the order of 2,4-DCP > 2CP > 3CP > 4CP, which was same as observed in the tertiary mixture.

The total chlorophenol degradation observed was higher in the presence of 2-CP as compared to 3-CP and 4-CP. The toxicity of chlorophenols was inversely proportional to the number of chloride ion substitutions. The toxicity of 2,4-DCP is higher compared to MCPs while the toxicity of MCPs decreases in the order of 3CP > 4CP > 2CP. In the present study, the defined mixed consortium showed higher degradation of 2,4-DCP as compared to MCPs. Some authors also reported the similar results. Zilouei et al., reported that higher biodegradation rate for 2,4-DCP and 2,4,6-TCP compared to 4-CP and 2-CP. The order of removal observed was increased in the order of TCP > DCP > 4CP > 2CP. The easily degradable 2-CP was observed to remove at the slowest rate. However, the exact mechanism involved was not understood (Zilouei et al. 2006). However, some studies reported that the degradation of various chlorophenols was not related to the chloride ion substitution pattern and explained that this phenomenon were due to metabolic pathway of the isolated strains (Yang et al. 2005).

The biodegradation of 2,4-DCP and total chlorophenol degradation rate was increased in the presence of MCPs, particularly for 2-CP. This phenomenon can be explained as the enzymes induced in the presence of 2-CP also facilitate the degradation of 2,4-DCP due to the molecular structural similarity between them in terms of the position of chloride ion. Both the MCPs and 2,4-DCP degradations proceed via chlorocatechol pathway. Another explanation for higher total chlorophenols removal is that the presence of MCPs contributes to biomass growth. The microorganism utilizes different bioenergetic strategy depending on the toxicity of the compounds. Papazi and Kotzabasis have also reported the similar results. They reported that the biodegradation of DCP congeners 2,3-DCP, 2,5-DCP and 3,4-DCP (higher toxicity, one *meta*-substitution) was higher compared to DCP congeners 2,4-DCP and 2,6-DCP (lower toxicity, no *meta*-substitution) and the corresponding MCPs (Papazi and Kotzabasis 2013). They have reported that in the presence of higher toxic compounds or when toxicity of compounds reaches a threshold level, the microorganism gives more energy to toxicity removal or biodegradation than to biomass growth. In the present study, a combination of 3-CP and 2,4-DCP has higher toxicity as compared to other mixture and for this mixture the biomass growth observed was very low and hence the overall degradation rate. While the presence of 2-CP leads to higher degradation rate by contributing to higher biomass growth as observed in previous batch studies explaining the lower degradation of 2-CP alone as compared to 3-CP and 4-CP.

Industrial effluents and contaminated environmental sites mostly contain the mixture of chlorophenols and other aromatic compounds. Interaction among these recalcitrant compounds is complex and has a great influence on the biodegradation of chlorophenols due to their toxicity, competition, molecular structure and enzyme expression (Sahinkaya and Dilek 2006b). Wang et al. (2014) reported that phenol could induce the enzyme required for 4-CP biodegradation in *P. putida* LY1. The result showed that the strain *P. putida* LY1 could not grow on 4-CP as sole carbon source. Co-metabolic study showed that at high phenol to 4-CP ratio, phenol was first transferred to metabolites that could be utilized by the bacteria for as growth substrate (Wang et al. 2014). Kim and Hao (1999) reported that critical ratio between phenol and chlorophenol was necessary for complete biodegradation of 3-CP and 4-CP and below this ratio, both 3-CP and 4-CP showed partial degradation (Kim and Hao 1999). Here, the strains utilize the phenol as a growth substrate and the enzymes induced by growth substrate acts on the non-growth substrate and add alteration to it or partially degrade the compound of interest. These induced enzymes do not guarantee the complete degradation of the secondary substrate.

In this study, the isolated mixed consortium showed excellent efficiency in degradation of the mixture of chlorophenols. All the four isolated strains were not able to degrade 3-CP and 4-CP individually except *Bacillus cereus* 3YS which had shown degradation at a lower concentration only (data not shown). But when all the four strains were mixed to form the bacterial consortium, they have shown good tolerance and degradation of 3-CP and 4-CP up to higher concentration. Kim et al., reported the complete biodegradation of phenol, 4-chlorophenol, and 2,4,6-trichlorophenol mixture by a defined mixed culture of *P. testosteroni* CPW301 and *P. solanacearum* TCP114 (Kim et al. 2002). Bae et al. isolated two different pure cultures which were able to degrade only selective substrate. *Pseudomonas* sp. TCP114 was able to degrade 2,4,6-TCP and phenol while *Arthrobacter* sp. CPR706 was only able to degrade 4-CP. When two bacteria were mixed, the resulting defined consortium was able to degrade all three chlorophenols simultaneously (Bae et al. 1997). Single bacterial strain can degrade the toxic compounds completely if provided the feasible environment and presence of primary growth substrate. However, sometimes pure strain does not possess or express all the enzymes required for complete mineralization of toxic compounds. They only express the enzymes that act on parent compounds and produce the intermediate metabolites. These intermediate metabolites remain in the medium unutilized because of the lack of enzymes required in the metabolic pathway. In addition, use of pure strains in the in situ environment is impractical as the dominance of other strains over the special strains that are better fitted for the degradation of the target compound.

## Conclusions

The defined mixed microbial consortium showed excellent degradation efficiency for monochlorophenols and 2,4-dichlorophenols alone and in the mixture. The consortium was able to utilize all three monochlorophenols efficiently up to 400 mg L^−1^ concentration. The metabolite analysis indicates that the consortium follows the *ortho*- as well as *meta*-cleavage pathways. Multi-substrate degradation study showed that the defined consortium was able to utilize MCPs and 2,4-DCP simultaneously and that the presence of MCPs leads to increased degradation of 2,4-DCP and total chlorophenols. Three main phenomena can be explained. First, the enzyme induced in the presence of MCPs facilitates the degradation of 2,4-DCP. Second, the presence of MCPs especially 2-CP in this case, leads to higher removal rate by contributing to higher biomass growth. Third, defined microbial consortium where different microorganisms act in a coordinated way to degrade specific recalcitrant compounds. The results showed that defined mixed consortium could utilize wide range of recalcitrant compounds individually as well as in mixture as compared to pure strains and more suited for in situ bioremediation. In the present study, the ability of the defined mixed consortium for degradation of a mixture of chlorophenols shows its application for wastewater treatment and in situ bioremediation.

## References

[CR1] Alexander M (1999). Biodegradation and Bioremediation.

[CR2] Andrews JF (1968). A mathematical model for the continuous culture of microorganisms utilizing inhibitory substrates. Biotechnol Bioeng.

[CR3] Arnow LE (1937). Colorimetric determination of the components of 3,4-dihydroxyphenylalaninetyrosine mixtures. J Biol Chem.

[CR4] Arora P, Bae H (2014). Bacterial degradation of chlorophenols and their derivatives. Microb Cell Fact.

[CR5] ATSDR (1999) Toxicological profile for Chlorophenols. US Department of Health and Human Services, Atlanta, GA

[CR6] ATSDR (2015) Comprehensive environmental response, compensation, and liability act (CERCLA), priority list of hazardous substances

[CR7] Bae HS, Lee JM, Lee S-T (1997). Biodegradation of the mixture of 2,4,6-trichlorophenol, 4-chiorophenol, and phenol by a defined mixed culture. J Gen Appl Microbiol.

[CR8] Baggi G, Andreoni V, Bernasconi S, Cavalca L, Zangrossi M (2002). Co-metabolic degradation of mixtures of monochlorophenols by phenol-degrading microorganisms. Ann Microbiol.

[CR9] Bartels I, Knackmuss H-J, Reineke W (1984). Suicide inactivation of catechol 2,3-dioxygenase from *Pseudomonas putida* mt-2 by 3-Halocatechols. Appl Environ Microbiol.

[CR10] De Los Cobos-Vasconcelos D, Santoyo-Tepole F, Juárez-Ramírez C, Ruiz-Ordaz N, Galíndez-Mayer CJJ (2006). Cometabolic degradation of chlorophenols by a strain of Burkholderia in fed-batch culture. Enzyme Microbial Technol.

[CR11] Durruty I, Okada E, González J, Murialdo S (2011). Multisubstrate monod kinetic model for simultaneous degradation of chlorophenol mixtures. Biotechnol Bioprocess Eng.

[CR12] El-Sayed WS, Ismaeil M, El-Beih F (2009) Isolation of 4-chlorophenol-degrading bacteria, *Bacillus subtilis* OS1 and *Alcaligenes* sp. OS2 from petroleum oil-contaminated soil and characterization of its catabolic pathway. Aust J Basic Appl Sci 3(2):776–789

[CR13] Farrell A, Quilty B (1999). Degradation of mono-chlorophenols by a mixed microbial community via a *meta*-cleavage pathway. Biodegradation.

[CR14] Field J, Sierra-Alvarez R (2008). Microbial degradation of chlorinated phenols. Rev Environ Sci Bio/Technol.

[CR15] Goswami M, Shivaraman N, Singh R (2002). Kinetics of chlorophenol degradation by benzoate-induced culture of *Rhodococcus erythropolis* M1. World J Microbiol Biotechnol.

[CR16] Häggblom M (1990). Mechanisms of bacterial degradation and transformation of chlorinated monoaromatic compounds. J Basic Microbiol.

[CR17] Herrera Y, Okoh A, Alvarez L, Robledo N, Trejo-Hernández M (2008). Biodegradation of 2,4-dichlorophenol by a Bacillus consortium. World J Microbiol Biotechnol.

[CR18] Kargi F, Eker S (2005). Kinetics of 2,4-dichlorophenol degradation by *Pseudomonas putida* CP1 in batch culture. Int Biodeterior Biodegrad.

[CR19] Karigar CS, Rao SS (2011). Role of microbial enzymes in the bioremediation of pollutants: a review. Enzyme Res.

[CR20] Karn SK, Chakrabarty SK, Reddy MS (2010). Pentachlorophenol degradation by *Pseudomonas stutzeri* CL7 in the secondary sludge of pulp and paper mill. J Environ Sci (China).

[CR21] Kim MH, Hao OJ (1999). Cometabolic degradation of chlorophenols by Acinetobacter species. Water Res.

[CR22] Kim J-H, Oh K-K, Lee S-T, Kim S-W, Hong S-I (2002). Biodegradation of phenol and chlorophenols with defined mixed culture in shake-flasks and a packed bed reactor. Process Biochem.

[CR23] Klecka GM, Gibson DT (1981). Inhibition of catechol 2,3-dioxygenase from *Pseudomonas putida* by 3-chlorocatechol. Appl Environ Microbiol.

[CR24] Louie T, Webster C, Xun L (2002). Genetic and biochemical characterization of a 2,4,6-trichlorophenol degradation pathway in *Ralstonia eutropha* JMP134. J Bacteriol.

[CR25] Marihal AK, Jagadeesh KS, Sinha S (2009). Biodegradation of PCP by the rhizobacteria isolated from pentachlorophenol-tolerant crop species. Int J Civil Environ Eng.

[CR26] Murialdo SE, Fenoglio R, Haure PM, Gonzalez JF (2003). Degradation of phenol and chlorophenols by mixed and pure cultures. Water SA.

[CR27] Nordin K, Unell M, Jansson JK (2005). Novel 4-chlorophenol degradation gene cluster and degradation route via hydroxyquinol in Arthrobacter chlorophenolicus A6. Appl Environ Microbiol.

[CR28] Olaniran AO, Igbinosa EO (2011). Chlorophenols and other related derivatives of environmental concern: properties, distribution and microbial degradation processes. Chemosphere.

[CR29] Papazi A, Kotzabasis K (2013). “Rational” management of dichlorophenols biodegradation by the microalga *Scenedesmus obliquus*. PLoS One.

[CR30] Patel BP, Kumar A (2016). Biodegradation of 2,4-dichlorophenol by Bacillus endophyticus strain: optimization of experimental parameters using response surface methodology and kinetic study. Desalination Water Treat.

[CR31] Patel BP, Kumar A (2016). Optimization study for maximizing 2,4-dichlorophenol degradation by *Kocuria rhizophila* strain using response surface methodology and kinetic study. Desalination Water Treat.

[CR32] Sahinkaya E, Dilek FB (2006). Effect of biogenic substrate concentration on 4-chlorophenol degradation kinetics in sequencing batch reactors with instantaneous feed. J Hazard Mater.

[CR33] Sahinkaya E, Dilek FB (2006). Effect of biogenic substrate concentration on the performance of sequencing batch reactor treating 4-CP and 2,4-DCP mixtures. J Hazard Mater.

[CR34] Sahinkaya E, Dilek FB (2007). Effect of feeding time on the performance of a sequencing batch reactor treating a mixture of 4-CP and 2,4-DCP. J Environ Manage.

[CR35] Schmidt E, Hellwig M, Knackmuss HJ (1983). Degradation of chlorophenols by a defined mixed microbial community. Appl Environ Microbiol.

[CR36] Shen DS, Liu XW, Feng HJ (2005). Effect of easily degradable substrate on anaerobic degradation of pentachlorophenol in an upflow anaerobic sludge blanket (UASB) reactor. J Hazard Mater.

[CR37] Snyder CJ, Asghar M, Scharer JM, Legge RL (2006). Biodegradation kinetics of 2,4,6-trichlorophenol by an acclimated mixed microbial culture under aerobic conditions. Biodegradation.

[CR38] Solyanikova IP, Golovleva LA (2004). Bacterial degradation of chlorophenols: pathways, biochemical, and genetic aspects. J Environ Sci Health Part B.

[CR39] Tobajas M, Monsalvo VM, Mohedano AF, Rodriguez JJ (2012). Enhancement of cometabolic biodegradation of 4-chlorophenol induced with phenol and glucose as carbon sources by *Comamonas testosteroni*. J Environ Manage.

[CR40] Tomei MC, Annesini MC, Bussoletti S (2004). 4-Nitrophenol biodegradation in a sequencing batch reactor: kinetic study and effect of filling time. Water Res.

[CR41] Veenagayathri K, Vasudevan N (2011). *Ortho* and *meta* cleavage dioxygenases detected during the degradation of phenolic compounds by a moderately halophilic bacterial consortium. Int Res J Microbiol.

[CR42] Wang Q, Li Y, Li J, Wang Y, Wang C, Wang P (2014) Experimental and kinetic study on the cometabolic biodegradation of phenol and 4-chlorophenol by psychrotrophic *Pseudomonas putida* LY1. Environ Sci Pollut Res 1–9. doi:10.1007/s11356-014-3374-x 10.1007/s11356-014-3374-x 25091164

[CR43] Westmeier F, Rehm HJ (1987). Degradation of 4-chlorophenol in municipal wastewater by adsorptive immobilized *Alcaligenes* sp. A 7-2. Appl Microbiol Biotechnol.

[CR44] Wieser M, Eberspächer J, Vogler B, Lingens F (1994) Metabolism of 4-chlorophenol by *Azotobacter* sp. GP1: structure of the *meta* cleavage product of 4-chlorocatechol. FEMS Microbiol Lett 116 (1):73–78 10.1111/j.1574-6968.1994.tb06678.x 8132157

[CR45] Yang CF, Lee CM, Wang CC (2005). Degradation of chlorophenols using pentachlorophenol-degrading bacteria Sphingomonas chlorophenolica in a batch reactor. Curr Microbiol.

[CR46] Ziagova M, Liakopoulou-Kyriakides M (2007) Kinetics of 2,4-dichlorophenol and 4-Cl-m-cresol degradation by *Pseudomonas* sp. cultures in the presence of glucose. Chemosphere 68(5):921–927. doi:10.1016/j.chemosphere.2007.01.039 10.1016/j.chemosphere.2007.01.039 17328941

[CR47] Zilouei H, Guieysse B, Mattiasson B (2006). Biological degradation of chlorophenols in packed-bed bioreactors using mixed bacterial consortia. Process Biochem.

